# Supplementary material for: Seasonal availability of edible underground and aboveground carbohydrate resources to human foragers on the Cape south coast, South Africa
Source: PeerJ. 2016 Feb 18;4:e1679. doi: 10.7717/peerj.1679 (PMC4768670; doi:10.7717/peerj.1679)
Supplement: Supplemental Information 1 — Meta data for raw data file. [file peerj-04-1679-s001.docx]

|  |  |  |  |  |  |  |  |  |  |  |  |  |  |  |  |  |  |
| --- | --- | --- | --- | --- | --- | --- | --- | --- | --- | --- | --- | --- | --- | --- | --- | --- | --- |
| **Meta data for raw data file** | | |  |  |  |  |  |  |  |  |  |  |  |  |  |  |  |
|  |  |  |  |  |  |  |  |  |  |  |  |  |  |  |  |  |  |
| Column A: Indigenous edible plant species observed over a two year sample period in the four primary  vegetation types of the southern Cape, South Africa. | | | | | | | | | | |  |  |  |  |  |  |  |
| Each vegetation type is listed as species with underground storage organs followed by aboveground edible  species. | | | | | | | | |  |  |  |  |  |  |  |  |  |
|  |  |  |  |  |  |  |  |  |  |  |  |  |  |  |  |  |  |
| Columns B to S: raw data collection dates (rows 6, 20, 34, 61, 85, 96, 107 and 128),  julien days (rows 7, 21, 35, 62, 86, 97, 108 and 129) and counts for the various species observed  in the sample plots of the four primary vegetation types. | | | | | | | | | | | | | | | | | |
| Counts per species per vegetation type are of the total sample plot areas (3.6 hectare). | | | | | | |  |  |  |  |  |  |  |  |  |  |  |
| Species with underground storage organs were only counted when they were visible and viable  from a foraging perspective. | | | | | | | | | |  |  |  |  |  |  |  |  |
| Aboveground species were only counted when they were in their edible or ripe phase. | | | | | | |  |  |  |  |  |  |  |  |  |  |  |
