# Supplementary material for: Seasonal availability of edible underground and aboveground carbohydrate resources to human foragers on the Cape south coast, South Africa
Source: PeerJ. 2016 Feb 18;4:e1679. doi: 10.7717/peerj.1679 (PMC4768670; doi:10.7717/peerj.1679)
Supplement: Supplemental Information 3 — An index of all supplementary tables and figures. Phenological phase synchronicity is explained in detail and the methods which were employed in creating Fig. S3. References for the phenological phase synchronicity description. [file peerj-04-1679-s003.docx]

# Supplementary Materials Methods

**Seasonal availability of edible underground and aboveground carbohydrate resources to human foragers on the Cape south coast, South Africa**

J.C. De Vynck, R.M. Cowling , A.J. Potts and C.W. Marean

The supplementary materials contains the following:

- Table S1: Biophysical data for the four survey plots
- Table S2.1 to S2.8: Phenological diagrams of all species encountered in the four survey plots
- Table S3: Total species list of USOs and fruiting species (aboveground carbohydrate resources) and their acronyms (used in Fig. S3) encountered in the four survey plots
- Table S4: Species list per vegetation type of USOs and fruiting species (aboveground carbohydrate resources), and their acronyms, encountered in the four survey plots
- Fig S1: Climate diagrams for the four survey plots
- Fig. S2: Locations of the vegetation survey plots and the weather stations
- Fig. S3: Phenological phase synchronicity (specifically availability of edible carbohydrates) among plant species from the four survey plots
- References for Phenological phase synchronicity description

**Phenological phase synchronicity**

To investigate patterns of phenological phase synchronicity (i.e. phenological timing) among edible USOs and fruiting species (reported above), we used we used hierarchical clustering (Anderberg, 1973). Such clustering requires a dissimilarity matrix as the input. Dissimilarity was calculated as the sum of the absolute differences of relative visibility between two species (including site combinations, e.g. species 1 at site 1 versus species 1 at site 2, or species 1 at site 1 versus species 2 at site 1) for each survey date. Hierarchical clustering was performed using the *hclust* function (Pinheiro et al., 2012) and the averaging agglomeration (UPGMA) method in R version 2.15 (R Development Core Team, 2014). The following R code was used:

> hclust(obj,method="ave")

where obj is a distance object with pairwise distances between species. Pairwise distance was calculated as the sum of the absolute differences of the proportional visibility between two species across all survey periods within a plot.

Defining clusters was not performed using a strict dissimilarity threshold, but rather involved intuitive exploration of the phenological diagrams of different potential clusters while endeavouring to maintain cluster thresholds that were fairly similar. This multivariate analysis categorised seven phenological phases of availability for edible underground and aboveground carbohydrates across the plots (A to G; Fig. S2). Most phenological phases comprised a combination of underground and aboveground carbohydrate species as well as species found in at least three, often all four, vegetation types. Exceptions are the summer phenological phase (B), which consisted entirely of aboveground carbohydrate species predominantly from Limestone Fynbos, and the extended autumn-to-summer phenophase (D), which only has species with underground storage organs.

**References for Phenological phase synchronicity**

Anderberg MR. 1973. *Cluster analysis for applications* (No. OAS-TR-73-9). New Mexico: Office of the Assistent for Study Support Kirtland AFB.

Pinheiro J, Bates D, DebRoy S, Sarkar D. 2012. R Development Core Team. nlme: Linear and nonlinear mixed effects models, 2012. *URL http://CRAN. R-project. org/package= nlme. R package version*, 3-1.

R Development Core Team. 2014. R: A language and environment for statistical computing. Vienna, Austria: R Foundation for Statistical computing, ISBN 3-900051-07-0, URL http://www.R-project.org
