# Supplementary material for: Seasonal availability of edible underground and aboveground carbohydrate resources to human foragers on the Cape south coast, South Africa
Source: PeerJ. 2016 Feb 18;4:e1679. doi: 10.7717/peerj.1679 (PMC4768670; doi:10.7717/peerj.1679)
Supplement: Supplemental Information 4 — Biophysical information for the four study sites. [file peerj-04-1679-s004.docx]

Table S1: Biophysical information for the four study sites.

|  | Strandveld | Limestone Fynbos | Sand Fynbos | Renosterveld |
| --- | --- | --- | --- | --- |
| Nature reserve | Gouritz Mouth Local Authority Nature Reserve | Pauline Bohnen Local Authority Nature Reserve | Rein’s Private Nature Reserve | Werner Frehze Local Authority Nature Reserve |
| Plot coordinates | S34̊ 21′17.38″  E21̊ 52′28.65″ | S34̊ 21′55.62″  E21̊ 25′26.00″ | S34̊ 20′40.87″S  E21̊ 45′59.80″E | S34̊ 06′58.25″  21̊ 14′59.70″E |
| MAP (mm) | 352 | 510 | 352 | 378 |
| Soil description | Deep alkaline sand; moderately fertile | Shallow, alkaline sand overlying limestone; infertile | Deep, leached, acid sand: infertile | Shallow, slightly acid loam overlying clayey sub-soil; moderately fertile |
| Dominant species | *Cassine peragua*  (Celastraceae)  *Eriocephalus africanus*  (Asteraceae)  *Osteospermum moniliferum*  (Asteraceae)  *Rhoicissus digitata*  (Vitaceae)  *Sideroxylon inerme*  (Sapotaceae)  *Zygophyllum morgsana*  (Zygophyllaceae) | *Erica spectabilis*  (Ericaceae)  *Ischyrolepis*  *leptocladus*  (Restionaceae)  *Leucadendron*  *meridianum*  (Proteaceae)  *Metalasia muricata* (Asteraceae)  *Stoebe muirrii*  (Asteraceae)  *Thamnochortus*  *muirrii*  (Restionaceae) | *Cliffortia illicifolia*  (Rosaceae)  *Erica dispar*  (Ericaceae)  *Leucadendron eucalyptifolium*  (Proteaceae)  *Leucospermum praecox*  (Proteaceae)  *Protea susannae*  (Proteaceae)  *Thamnochortus insignis*  (Restionaceae)  *Watsonia fourcadei*  (Iridaceae) | *Aloe ferox*  (Asphodelaceae) *Elytropappus rhinocerotis*  (Asteraceae)  *Ehrharta calycina* (Poaceae)  *Eriocephalus africanus*  (Asteraceae) *Metalasia pungens*  (Asteraceae) *Themeda triandra*  (Poaceae) |
