# Supplementary material for: Seasonal availability of edible underground and aboveground carbohydrate resources to human foragers on the Cape south coast, South Africa
Source: PeerJ. 2016 Feb 18;4:e1679. doi: 10.7717/peerj.1679 (PMC4768670; doi:10.7717/peerj.1679)
Supplement: Supplemental Information 5 — Phenology diagrams for plant species with Underground Storage Organs (USOs) and for fruiting plant species (aboveground carbohydrate resources) of the four primary vegetation types of the south Cape lowlands to coastal margin. [file peerj-04-1679-s005.docx]

**Phenology diagrams**

This section includes phenological diagrams of plant species with edible underground storage organs (USOs) (Tables S2.1 to S2.4) and fruiting species (aboveground carbohydrate resources) (Tables S2.5 to S2.8) encountered in the phenology survey of the four primary vegetation types of the southern Cape lowlands to coastal margin. These surveys were conducted every six weeks over a two year period. All species are listed with their respective family names. The colour keys indicate the **average** annual phenological phases derived from the two year survey. For USOs, all phenological phases, apart from black (‘Not visible’), are visible and therefore procurable by a human forager. For fruiting species, the blue colour key (‘Edible/ripe fruit’) is the only phenological phase important for human foragers as this is the only phase with edible components on the respective plants.

The following colours are used to represent the different phenological phase in the tables below:

|  | Dry leaves |
| --- | --- |
|  | Green leaves |
|  | Flowering |
|  | Ripe/edible fruit |
|  | Not visible |

The “Dry leaves”, “Green leaves” and “Flowering” phenological phases are shown on their own lines. However, for the plant species with underground storage organs (USOs), black shading is used over all three lines per species to emphasize when carbohydrates are not indirectly visible (i.e. there are no aboveground indicators of USOs). For fruiting plant species (aboveground carbohydrate resources), black shading is used in the same line as “Ripe/edible fruit” to emphasize when the edible components of the plants are not available or directly visible (i.e. there are no aboveground carbohydrates such as fruit).

Table S2.1. Phenology diagrams for plant species with underground storage organs (USOs) in the **Limestone Fynbos** plot.

|  | J | F | M | A | M | J | J | A | S | O | N | D |
| --- | --- | --- | --- | --- | --- | --- | --- | --- | --- | --- | --- | --- |
| *Babiana patula* |  |  |  |  |  |  |  |  |  |  |  |  |
| (Iridaceae) |  |  |  |  |  |  |  |  |  |  |  |  |
|  |  |  |  |  |  |  |  |  |  |  |  |  |
| *Cyanella lutea* |  |  |  |  |  |  |  |  |  |  |  |  |
| (Tecophilaeaceae) |  |  |  |  |  |  |  |  |  |  |  |  |
|  |  |  |  |  |  |  |  |  |  |  |  |  |
| *Cyphia digitata* |  |  |  |  |  |  |  |  |  |  |  |  |
| (Campanulaceae) |  |  |  |  |  |  |  |  |  |  |  |  |
|  |  |  |  |  |  |  |  |  |  |  |  |  |
| *Ferraria crispa* |  |  |  |  |  |  |  |  |  |  |  |  |
| (Iridaceae) |  |  |  |  |  |  |  |  |  |  |  |  |
|  |  |  |  |  |  |  |  |  |  |  |  |  |
| *Freesia alba* |  |  |  |  |  |  |  |  |  |  |  |  |
| (Iridaceae) |  |  |  |  |  |  |  |  |  |  |  |  |
|  |  |  |  |  |  |  |  |  |  |  |  |  |
| *Gladiolus cunonius* |  |  |  |  |  |  |  |  |  |  |  |  |
| (Iridaceae) |  |  |  |  |  |  |  |  |  |  |  |  |
|  |  |  |  |  |  |  |  |  |  |  |  |  |
| *Gladiolus exilis* |  |  |  |  |  |  |  |  |  |  |  |  |
| (Iridaceae) |  |  |  |  |  |  |  |  |  |  |  |  |
|  |  |  |  |  |  |  |  |  |  |  |  |  |
| *Gladiolus floribundus* |  |  |  |  |  |  |  |  |  |  |  |  |
| (Iridaceae) |  |  |  |  |  |  |  |  |  |  |  |  |
|  |  |  |  |  |  |  |  |  |  |  |  |  |
| *Gladiolus virescens* |  |  |  |  |  |  |  |  |  |  |  |  |
| (Iridaceae) |  |  |  |  |  |  |  |  |  |  |  |  |
|  |  |  |  |  |  |  |  |  |  |  |  |  |
| *Hesperantha falcata* |  |  |  |  |  |  |  |  |  |  |  |  |
| (Iridaceae) |  |  |  |  |  |  |  |  |  |  |  |  |
|  |  |  |  |  |  |  |  |  |  |  |  |  |
| *Ixia micrandra* |  |  |  |  |  |  |  |  |  |  |  |  |
| (Iridaceae) |  |  |  |  |  |  |  |  |  |  |  |  |
|  |  |  |  |  |  |  |  |  |  |  |  |  |
| *Moraea fugax* |  |  |  |  |  |  |  |  |  |  |  |  |
| (Iridaceae) |  |  |  |  |  |  |  |  |  |  |  |  |
|  |  |  |  |  |  |  |  |  |  |  |  |  |

|  | J | F | M | A | M | J | J | A | S | O | N | D |
| --- | --- | --- | --- | --- | --- | --- | --- | --- | --- | --- | --- | --- |
| *Pelargonium dipetalum* |  |  |  |  |  |  |  |  |  |  |  |  |
| (Geraniaceae) |  |  |  |  |  |  |  |  |  |  |  |  |
|  |  |  |  |  |  |  |  |  |  |  |  |  |
| *Pelargonium lobatum* |  |  |  |  |  |  |  |  |  |  |  |  |
| (Geraniaceae) |  |  |  |  |  |  |  |  |  |  |  |  |
|  |  |  |  |  |  |  |  |  |  |  |  |  |
| *Pelargonium triste* |  |  |  |  |  |  |  |  |  |  |  |  |
| (Geraniaceae) |  |  |  |  |  |  |  |  |  |  |  |  |
|  |  |  |  |  |  |  |  |  |  |  |  |  |
| *Romulea rosea* |  |  |  |  |  |  |  |  |  |  |  |  |
| (Iridaceae) |  |  |  |  |  |  |  |  |  |  |  |  |
|  |  |  |  |  |  |  |  |  |  |  |  |  |
| *Trachyandra ciliata* |  |  |  |  |  |  |  |  |  |  |  |  |
| (Asphodelaceae) |  |  |  |  |  |  |  |  |  |  |  |  |
|  |  |  |  |  |  |  |  |  |  |  |  |  |
| *Trachyandra revoluta* |  |  |  |  |  |  |  |  |  |  |  |  |
| (Asphodelaceae) |  |  |  |  |  |  |  |  |  |  |  |  |
|  |  |  |  |  |  |  |  |  |  |  |  |  |
| *Tritonia squalida* |  |  |  |  |  |  |  |  |  |  |  |  |
| (Iridaceae) |  |  |  |  |  |  |  |  |  |  |  |  |
|  |  |  |  |  |  |  |  |  |  |  |  |  |
| *Watsonia fergusoniae* |  |  |  |  |  |  |  |  |  |  |  |  |
| (Iridaceae) |  |  |  |  |  |  |  |  |  |  |  |  |
|  |  |  |  |  |  |  |  |  |  |  |  |  |

Table S2.2. Phenology diagrams for plant species with underground storage organs (USOs) in the **Renosterveld** plot.

|  | J | F | M | A | M | J | J | A | S | O | N | D |
| --- | --- | --- | --- | --- | --- | --- | --- | --- | --- | --- | --- | --- |
| *Babiana patulla* |  |  |  |  |  |  |  |  |  |  |  |  |
| (Iridaceae) |  |  |  |  |  |  |  |  |  |  |  |  |
|  |  |  |  |  |  |  |  |  |  |  |  |  |
| *Cyphia digitata* |  |  |  |  |  |  |  |  |  |  |  |  |
| (Campanulaceae) |  |  |  |  |  |  |  |  |  |  |  |  |
|  |  |  |  |  |  |  |  |  |  |  |  |  |
| *Freesia caryophyllacea* |  |  |  |  |  |  |  |  |  |  |  |  |
| (Iridaceae) |  |  |  |  |  |  |  |  |  |  |  |  |
|  |  |  |  |  |  |  |  |  |  |  |  |  |
| *Freesia leichtlinii* |  |  |  |  |  |  |  |  |  |  |  |  |
| (Iridaceae) |  |  |  |  |  |  |  |  |  |  |  |  |
|  |  |  |  |  |  |  |  |  |  |  |  |  |
| *Pelargonium lobatum* |  |  |  |  |  |  |  |  |  |  |  |  |
| (Geraniaceae) |  |  |  |  |  |  |  |  |  |  |  |  |
|  |  |  |  |  |  |  |  |  |  |  |  |  |
| *Pelargonium repaceum* |  |  |  |  |  |  |  |  |  |  |  |  |
| (Geraniaceae) |  |  |  |  |  |  |  |  |  |  |  |  |
|  |  |  |  |  |  |  |  |  |  |  |  |  |
| *Watsonia alletroides* |  |  |  |  |  |  |  |  |  |  |  |  |
| (Iridaceae) |  |  |  |  |  |  |  |  |  |  |  |  |
|  |  |  |  |  |  |  |  |  |  |  |  |  |
| *Watsonia meriana* |  |  |  |  |  |  |  |  |  |  |  |  |
| (Iridaceae) |  |  |  |  |  |  |  |  |  |  |  |  |
|  |  |  |  |  |  |  |  |  |  |  |  |  |

Table S2.3. Phenology diagrams for plant species with underground storage organs (USOs) in the **Sand Fynbos** plot.

|  | J | F | M | A | M | J | J | A | S | O | N | D |
| --- | --- | --- | --- | --- | --- | --- | --- | --- | --- | --- | --- | --- |
| *Gladiolus guthriei* |  |  |  |  |  |  |  |  |  |  |  |  |
| (Iridaceae) |  |  |  |  |  |  |  |  |  |  |  |  |
|  |  |  |  |  |  |  |  |  |  |  |  |  |
| *Gladiolus rogersii* |  |  |  |  |  |  |  |  |  |  |  |  |
| (Iridaceae) |  |  |  |  |  |  |  |  |  |  |  |  |
|  |  |  |  |  |  |  |  |  |  |  |  |  |
| *Pelargonium triste* |  |  |  |  |  |  |  |  |  |  |  |  |
| (Geraniaceae) |  |  |  |  |  |  |  |  |  |  |  |  |
|  |  |  |  |  |  |  |  |  |  |  |  |  |
| *Trachyandra revoluta* |  |  |  |  |  |  |  |  |  |  |  |  |
| (Asphodelaceae) |  |  |  |  |  |  |  |  |  |  |  |  |
|  |  |  |  |  |  |  |  |  |  |  |  |  |
| *Watsonia fourcadei* |  |  |  |  |  |  |  |  |  |  |  |  |
| (Iridaceae) |  |  |  |  |  |  |  |  |  |  |  |  |
|  |  |  |  |  |  |  |  |  |  |  |  |  |

Table S2.4. Phenology diagrams for plant species with underground storage organs (USOs) in the **Strandveld** plot.

|  | J | F | M | A | M | J | J | A | S | O | N | D |
| --- | --- | --- | --- | --- | --- | --- | --- | --- | --- | --- | --- | --- |
| *Babiana patulla* |  |  |  |  |  |  |  |  |  |  |  |  |
| (Iridaceae) |  |  |  |  |  |  |  |  |  |  |  |  |
|  |  |  |  |  |  |  |  |  |  |  |  |  |
| *Chasmanthe aethiopica* |  |  |  |  |  |  |  |  |  |  |  |  |
| (Iridaceae) |  |  |  |  |  |  |  |  |  |  |  |  |
|  |  |  |  |  |  |  |  |  |  |  |  |  |
| *Cyanella lutea* |  |  |  |  |  |  |  |  |  |  |  |  |
| (Tecophilaeaceae) |  |  |  |  |  |  |  |  |  |  |  |  |
|  |  |  |  |  |  |  |  |  |  |  |  |  |
| *Cyphia digitata* |  |  |  |  |  |  |  |  |  |  |  |  |
| (Campanulaceae) |  |  |  |  |  |  |  |  |  |  |  |  |
|  |  |  |  |  |  |  |  |  |  |  |  |  |
| *Ferraria crispa* |  |  |  |  |  |  |  |  |  |  |  |  |
| (Iridaceae) |  |  |  |  |  |  |  |  |  |  |  |  |
|  |  |  |  |  |  |  |  |  |  |  |  |  |
| *Freesia alba* |  |  |  |  |  |  |  |  |  |  |  |  |
| (Iridaceae) |  |  |  |  |  |  |  |  |  |  |  |  |
|  |  |  |  |  |  |  |  |  |  |  |  |  |
| *Gladiolus floribundus* |  |  |  |  |  |  |  |  |  |  |  |  |
| (Iridaceae) |  |  |  |  |  |  |  |  |  |  |  |  |
|  |  |  |  |  |  |  |  |  |  |  |  |  |
| *Oxalis pes-caprae* |  |  |  |  |  |  |  |  |  |  |  |  |
| (Oxalidaceae) |  |  |  |  |  |  |  |  |  |  |  |  |
|  |  |  |  |  |  |  |  |  |  |  |  |  |
| *Pelargonium lobatum* |  |  |  |  |  |  |  |  |  |  |  |  |
| (Geraniaceae) |  |  |  |  |  |  |  |  |  |  |  |  |
|  |  |  |  |  |  |  |  |  |  |  |  |  |
| *Pelargonium triste* |  |  |  |  |  |  |  |  |  |  |  |  |
| (Geraniaceae) |  |  |  |  |  |  |  |  |  |  |  |  |
|  |  |  |  |  |  |  |  |  |  |  |  |  |
| *Romulea rosea* |  |  |  |  |  |  |  |  |  |  |  |  |
| (Iridaceae) |  |  |  |  |  |  |  |  |  |  |  |  |
|  |  |  |  |  |  |  |  |  |  |  |  |  |
| *Trachyandra ciliata* |  |  |  |  |  |  |  |  |  |  |  |  |
| (Asphodelaceae) |  |  |  |  |  |  |  |  |  |  |  |  |
|  |  |  |  |  |  |  |  |  |  |  |  |  |

|  | J | F | M | A | M | J | J | A | S | O | N | D |
| --- | --- | --- | --- | --- | --- | --- | --- | --- | --- | --- | --- | --- |
| *Trachyandra revoluta* |  |  |  |  |  |  |  |  |  |  |  |  |
| (Asphodelaceae) |  |  |  |  |  |  |  |  |  |  |  |  |
|  |  |  |  |  |  |  |  |  |  |  |  |  |
| *Tritonia crocata* |  |  |  |  |  |  |  |  |  |  |  |  |
| (Iridaceae) |  |  |  |  |  |  |  |  |  |  |  |  |
|  |  |  |  |  |  |  |  |  |  |  |  |  |

Table S2.5. Phenology diagrams for fruiting plant species (aboveground carbohydrate resources) in the **Limestone Fynbos** plot.

|  | J | F | M | A | M | J | J | A | S | O | N | D |
| --- | --- | --- | --- | --- | --- | --- | --- | --- | --- | --- | --- | --- |
| *Astephanus triflorus* |  |  |  |  |  |  |  |  |  |  |  |  |
| (Apocynaceae) |  |  |  |  |  |  |  |  |  |  |  |  |
|  |  |  |  |  |  |  |  |  |  |  |  |  |
| *Carissa bispinosa* |  |  |  |  |  |  |  |  |  |  |  |  |
| (Apocynaceae) |  |  |  |  |  |  |  |  |  |  |  |  |
|  |  |  |  |  |  |  |  |  |  |  |  |  |
| *Carpobrotus acinaciformis* |  |  |  |  |  |  |  |  |  |  |  |  |
| (Aizoaceae) |  |  |  |  |  |  |  |  |  |  |  |  |
|  |  |  |  |  |  |  |  |  |  |  |  |  |
| *Carpobrotus edulis* |  |  |  |  |  |  |  |  |  |  |  |  |
| (Aizoaceae) |  |  |  |  |  |  |  |  |  |  |  |  |
|  |  |  |  |  |  |  |  |  |  |  |  |  |
| *Cassine tetragona* |  |  |  |  |  |  |  |  |  |  |  |  |
| (Celastraceae) |  |  |  |  |  |  |  |  |  |  |  |  |
|  |  |  |  |  |  |  |  |  |  |  |  |  |
| *Cynanchum obtusifolium* |  |  |  |  |  |  |  |  |  |  |  |  |
| (Apocynaceae) |  |  |  |  |  |  |  |  |  |  |  |  |
|  |  |  |  |  |  |  |  |  |  |  |  |  |
| *Euclea racemosa* |  |  |  |  |  |  |  |  |  |  |  |  |
| (Ebenaceae) |  |  |  |  |  |  |  |  |  |  |  |  |
|  |  |  |  |  |  |  |  |  |  |  |  |  |
| *Muraltia spinosa* |  |  |  |  |  |  |  |  |  |  |  |  |
| (Polygalaceae) |  |  |  |  |  |  |  |  |  |  |  |  |
|  |  |  |  |  |  |  |  |  |  |  |  |  |
| *Olea exasperata* |  |  |  |  |  |  |  |  |  |  |  |  |
| (Oleaceae) |  |  |  |  |  |  |  |  |  |  |  |  |
|  |  |  |  |  |  |  |  |  |  |  |  |  |
| *Osteospernum moniliferum* |  |  |  |  |  |  |  |  |  |  |  |  |
| (Asteraceae) |  |  |  |  |  |  |  |  |  |  |  |  |
|  |  |  |  |  |  |  |  |  |  |  |  |  |
| *Osyris compressa* |  |  |  |  |  |  |  |  |  |  |  |  |
| (Santalaceae) |  |  |  |  |  |  |  |  |  |  |  |  |
|  |  |  |  |  |  |  |  |  |  |  |  |  |
| *Searsia glauca* |  |  |  |  |  |  |  |  |  |  |  |  |
| (Anacardiaceae) |  |  |  |  |  |  |  |  |  |  |  |  |
|  |  |  |  |  |  |  |  |  |  |  |  |  |
| *Searsia lucida* |  |  |  |  |  |  |  |  |  |  |  |  |
| (Anacardiaceae) |  |  |  |  |  |  |  |  |  |  |  |  |
|  |  |  |  |  |  |  |  |  |  |  |  |  |
| *Sideroxylon inerme* |  |  |  |  |  |  |  |  |  |  |  |  |
| (Sapotaceae) |  |  |  |  |  |  |  |  |  |  |  |  |
|  |  |  |  |  |  |  |  |  |  |  |  |  |
| *Tetragonia decumbens* |  |  |  |  |  |  |  |  |  |  |  |  |
| (Aizoaceae) |  |  |  |  |  |  |  |  |  |  |  |  |
|  |  |  |  |  |  |  |  |  |  |  |  |  |
| *Trachyandra ciliata* |  |  |  |  |  |  |  |  |  |  |  |  |
| (Asphodelaceae) |  |  |  |  |  |  |  |  |  |  |  |  |
|  |  |  |  |  |  |  |  |  |  |  |  |  |
| *Zygophyllum morgsana* |  |  |  |  |  |  |  |  |  |  |  |  |
| (Zygophyllaceae) |  |  |  |  |  |  |  |  |  |  |  |  |
|  |  |  |  |  |  |  |  |  |  |  |  |  |

Table S2.6. Phenology diagrams for fruiting plant species (aboveground carbohydrate resources) in the **Renosterveld** plot.

|  | J | F | M | A | M | J | J | A | S | O | N | D |
| --- | --- | --- | --- | --- | --- | --- | --- | --- | --- | --- | --- | --- |
| *Carissa bispinosa* |  |  |  |  |  |  |  |  |  |  |  |  |
| (Apocynaceae) |  |  |  |  |  |  |  |  |  |  |  |  |
|  |  |  |  |  |  |  |  |  |  |  |  |  |
| *Diospyros dichrophylla* |  |  |  |  |  |  |  |  |  |  |  |  |
| (Ebenaceae) |  |  |  |  |  |  |  |  |  |  |  |  |
|  |  |  |  |  |  |  |  |  |  |  |  |  |
| *Microloma saggitatum* |  |  |  |  |  |  |  |  |  |  |  |  |
| (Asclepiadaceae) |  |  |  |  |  |  |  |  |  |  |  |  |
|  |  |  |  |  |  |  |  |  |  |  |  |  |
| *Muraltia spinosa* |  |  |  |  |  |  |  |  |  |  |  |  |
| (Polygalaceae) |  |  |  |  |  |  |  |  |  |  |  |  |
|  |  |  |  |  |  |  |  |  |  |  |  |  |
| *Osteospermum moniliferum* |  |  |  |  |  |  |  |  |  |  |  |  |
| (Asteraceae) |  |  |  |  |  |  |  |  |  |  |  |  |
|  |  |  |  |  |  |  |  |  |  |  |  |  |
| *Osyris compressa* |  |  |  |  |  |  |  |  |  |  |  |  |
| (Santalaceae) |  |  |  |  |  |  |  |  |  |  |  |  |
|  |  |  |  |  |  |  |  |  |  |  |  |  |
| *Searsia glauca* |  |  |  |  |  |  |  |  |  |  |  |  |
| (Anacardiaceae) |  |  |  |  |  |  |  |  |  |  |  |  |
|  |  |  |  |  |  |  |  |  |  |  |  |  |
| *Sideroxylon inerme* |  |  |  |  |  |  |  |  |  |  |  |  |
| (Sapotaceae) |  |  |  |  |  |  |  |  |  |  |  |  |
|  |  |  |  |  |  |  |  |  |  |  |  |  |

Table S2.7. Phenology diagrams for fruiting plant species (aboveground carbohydrate resources) in the **Sand Fynbos** plot.

|  | J | F | M | A | M | J | J | A | S | O | N | D |
| --- | --- | --- | --- | --- | --- | --- | --- | --- | --- | --- | --- | --- |
| *Carpobrotus edulis* |  |  |  |  |  |  |  |  |  |  |  |  |
| (Aizoaceae) |  |  |  |  |  |  |  |  |  |  |  |  |
|  |  |  |  |  |  |  |  |  |  |  |  |  |
| *Diospyros dichrophylla* |  |  |  |  |  |  |  |  |  |  |  |  |
| (Ebenaceae) |  |  |  |  |  |  |  |  |  |  |  |  |
|  |  |  |  |  |  |  |  |  |  |  |  |  |
| *Osteospernum moniliferum* |  |  |  |  |  |  |  |  |  |  |  |  |
| (Asteraceae) |  |  |  |  |  |  |  |  |  |  |  |  |
|  |  |  |  |  |  |  |  |  |  |  |  |  |
| *Searsia glauca* |  |  |  |  |  |  |  |  |  |  |  |  |
| (Anacardiaceae) |  |  |  |  |  |  |  |  |  |  |  |  |
|  |  |  |  |  |  |  |  |  |  |  |  |  |

Table S2.8. Phenology diagrams for fruiting plant species (aboveground carbohydrate resources) in the **Strandveld** plot.

|  | J | F | M | A | M | J | J | A | S | O | N | D |
| --- | --- | --- | --- | --- | --- | --- | --- | --- | --- | --- | --- | --- |
| *Carissa bispinosa* |  |  |  |  |  |  |  |  |  |  |  |  |
| (Apocynaceae) |  |  |  |  |  |  |  |  |  |  |  |  |
|  |  |  |  |  |  |  |  |  |  |  |  |  |
| *Carpobrotus acinaciformis* |  |  |  |  |  |  |  |  |  |  |  |  |
| (Aizoaceae) |  |  |  |  |  |  |  |  |  |  |  |  |
|  |  |  |  |  |  |  |  |  |  |  |  |  |
| *Cassine tetragona* |  |  |  |  |  |  |  |  |  |  |  |  |
| (Celastraceae) |  |  |  |  |  |  |  |  |  |  |  |  |
|  |  |  |  |  |  |  |  |  |  |  |  |  |
| *Muraltia spinosa* |  |  |  |  |  |  |  |  |  |  |  |  |
| (Polygalaceae) |  |  |  |  |  |  |  |  |  |  |  |  |
|  |  |  |  |  |  |  |  |  |  |  |  |  |
| *Olea exasperata* |  |  |  |  |  |  |  |  |  |  |  |  |
| (Oleaceae) |  |  |  |  |  |  |  |  |  |  |  |  |
|  |  |  |  |  |  |  |  |  |  |  |  |  |
| *Osteospermum moniliferum* |  |  |  |  |  |  |  |  |  |  |  |  |
| (Asteraceae) |  |  |  |  |  |  |  |  |  |  |  |  |
|  |  |  |  |  |  |  |  |  |  |  |  |  |
| *Schotia afra* |  |  |  |  |  |  |  |  |  |  |  |  |
| (Fabaceae) |  |  |  |  |  |  |  |  |  |  |  |  |
|  |  |  |  |  |  |  |  |  |  |  |  |  |
| *Searsia glauca* |  |  |  |  |  |  |  |  |  |  |  |  |
| (Anacardiaceae) |  |  |  |  |  |  |  |  |  |  |  |  |
|  |  |  |  |  |  |  |  |  |  |  |  |  |
| *Sideroxylon inerme* |  |  |  |  |  |  |  |  |  |  |  |  |
| (Sapotaceae) |  |  |  |  |  |  |  |  |  |  |  |  |
|  |  |  |  |  |  |  |  |  |  |  |  |  |
| *Tetragonia decumbens* |  |  |  |  |  |  |  |  |  |  |  |  |
| (Aizoaceae) |  |  |  |  |  |  |  |  |  |  |  |  |
|  |  |  |  |  |  |  |  |  |  |  |  |  |
| *Trachyandra ciliata* |  |  |  |  |  |  |  |  |  |  |  |  |
| (Asphodelaceae) |  |  |  |  |  |  |  |  |  |  |  |  |
|  |  |  |  |  |  |  |  |  |  |  |  |  |
| *Zygophyllum morgsana* |  |  |  |  |  |  |  |  |  |  |  |  |
| (Zygophyllaceae) |  |  |  |  |  |  |  |  |  |  |  |  |
|  |  |  |  |  |  |  |  |  |  |  |  |  |
