# Supplementary material for: Seasonal availability of edible underground and aboveground carbohydrate resources to human foragers on the Cape south coast, South Africa
Source: PeerJ. 2016 Feb 18;4:e1679. doi: 10.7717/peerj.1679 (PMC4768670; doi:10.7717/peerj.1679)
Supplement: Supplemental Information 6 — Total species list of USOs and fruiting species (aboveground carbohydrate resources) and their acronyms (used in Fig. S3) encountered in the phenology survey of plots within four vegetation types of the southern Cape lowlands to coastal margin. [file peerj-04-1679-s006.docx]

Table S3. Total species list of USOs and fruiting species (aboveground carbohydrate resources) and their acronyms (used in Fig. S3) encountered in the phenology survey of plots within four vegetation types of the southern Cape lowlands to coastal margin.

| **USO species** | |  | **Fruiting & other species** | |
| --- | --- | --- | --- | --- |
| BAB PAT | *Babiana patula* |  | AST TRI | *Astephanus triflorus* (fruit) |
| CHA AET | *Chasmanthe aethiopica* |  | CAR BIS | *Carissa bispinosa* (fruit) |
| CYA LUT | *Cyanella lutea* |  | CAR ACI | *Carpobrotus acinaciformis* (fruit) |
| CYP DIG | *Cyphia digitata* |  | CAR EDU | *Carpobrotus edulis* (fruit) |
| FER CRI | *Ferraria crispa* |  | CAS TET | *Cassine tetragona* (fruit) |
| FRE ALB | *Freesia alba* |  | CYN OBT | *Cynanchum obtusifolium* (fruit) |
| FRE CAR | *Freesia caryophyllacea* |  | DIO DIC | *Diospyros dichrophylla* (fruit) |
| FRE LEI | *Freesia leichtlinii* |  | EUC RAC | *Euclea racemosa* (fruit) |
| GLA CUN | *Gladiolus cunonius* |  | MIC SAG | *Microloma sagittatum* (fruit) |
| GLA EXI | *Gladiolus exilis* |  | MUR SPI | *Muraltia spinosa* (fruit) |
| GLA FLO | *Gladiolus floribundus* |  | OLE EXA | *Olea exasperata* (fruit) |
| GLA GUT | *Gladiolus guthriei* |  | OST MON | *Osteospermum moniliferum* (fruit) |
| GLA ROG | *Gladiolus rogersii* |  | OSY COM | *Osyris compressa* (fruit, seed) |
| GLA VIR | *Gladiolus virescens* |  | SCH AFR | *Schotia afra* (seed) |
| HES FAL | *Hesperantha falcata* |  | SEA GLA | *Searsia glauca* (fruit) |
| IXI MIC | *Ixia micrandra* |  | SEA LUC | *Searsia lucida* (fruit) |
| MOR FUG | *Moraea fugax* |  | SID INE | *Sideroxylon inerme* (fruit) |
| OXA PES | *Oxalis pes-caprae* |  | TET DEC | *Tetragonia decumbens* (veg.) |
| PEL DIP | *Pelargonium dipetalum* |  | TRA CIL | *Trachyandra ciliata* (veg.) |
| PEL LOB | *Pelargonium lobatum* |  | TRA REV | *Trachyandra revoluta* (veg.) |
| PEL REP | *Pelargonium rapaceum* |  | ZYG MOR | *Zygophyllum morgsana* (seed) |
| PEL TRI | *Pelargonium triste* |  |  |  |
| RHO DIG | *Rhoicissus digitata* |  |  |  |
| ROM ROS | *Romulea rosea* |  |  |  |
| TRA CIL | *Trachyandra ciliata* |  |  |  |
| TRA REV | *Trachyandra revoluta* |  |  |  |
| TRI CRO | *Tritonia crocata* |  |  |  |
| TRI SQU | *Tritonia squalida* |  |  |  |
| WAT ALL | *Watsonia aletroides* |  |  |  |
| WAT FER | *Watsonia fergusoniae* |  |  |  |
| WAT FOU | *Watsonia fourcadei* |  |  |  |
| WAT MER | *Watsonia meriana* |  |  |  |
