# Supplementary material for: Seasonal availability of edible underground and aboveground carbohydrate resources to human foragers on the Cape south coast, South Africa
Source: PeerJ. 2016 Feb 18;4:e1679. doi: 10.7717/peerj.1679 (PMC4768670; doi:10.7717/peerj.1679)
Supplement: Supplemental Information 7 — Species list of USOs and fruiting species (aboveground carbohydrate resources), and their acronyms, encountered in the phenology survey list of plots within four dominant vegetation types in the southern Cape lowlands to coastal margin. Acronyms relate to those used in Fig. S3. [file peerj-04-1679-s007.docx]

Table S4. Species list of USOs and fruiting species (aboveground carbohydrate resources), and their acronyms, encountered in the phenology survey list of plots within four dominant vegetation types in the southern Cape lowlands to coastal margin. Acronyms relate to those used in Fig. S3.

| **USO species** | Acronym |  | **Fruiting species** (aboveground carbohydrates) | Acronym |
| --- | --- | --- | --- | --- |
| **Strandveld** |  |  | **Strandveld** |  |
| *Babiana patulla* | BAB PAT |  | *Carissa bispinosa* (fruit) | CAR BIS |
| *Chasmanthe aetiopica* | CHA AET |  | *Carpobrotus acinaciformis* (fruit) | CAR ACI |
| *Cyanella lutea* | CYA LUT |  | *Cassine tetragona* (fruit) | CAS TET |
| *Cyphia digitata* | CYP DIG |  | *Muraltia spinosa* (fruit) | MUR SPI |
| *Ferraria crispa* | FER CRI |  | *Olea exasperata* (fruit) | OLE EXA |
| *Freesia alba* | FRE ALB |  | *Osteospermum moniliferum (*fruit) | OST MON |
| *Gladiolus floribundus* | GLA FLO |  | *Schotia afra* (seed) | SCH AFR |
| *Oxalis pes-caprae* | OXA PES |  | *Searsia glauca* (fruit) | SEA GLA |
| *Pelargonium lobatum* | PEL LOB |  | *Sideroxylon inerme* (fruit) | SID INE |
| *Pelargonium triste* | PEL TRI |  | *Tetragonia decumbens* (veg.) | TET DEC |
| *Rhoicissus digitata* | RHO DIG |  | *Trachyandra ciliata* (veg.) | TRA CIL |
| *Romulea rosea* | ROM ROS |  | *Trachyandra revoluta* (veg.) | TRA REV |
| *Trachyandra ciliata* | TRA CIL |  | *Zygophyllum morgsana* (seed) | ZYG MOR |
| *Trachyandra revoluta* | TRA REV |  |  |  |
| *Tritonia crocata* | TRI CRO |  |  |  |
| **Limestone Fynbos** |  |  | **Limestone Fynbos** |  |
| *Babiana patulla* | BAB PAT |  | *Astephanus triflorus* (veg.) | AST TRI |
| *Cyanella lutea* | CYA LUT |  | *Carissa bispinosa* (fruit) | CAR BIS |
| *Cyphia digitata* | CYP DIG |  | *Carpobrotus acinaciformis* (fruit) | CAR ACI |
| *Ferraria crispa* | FER CRI |  | *Carpobrotus edulis* (fruit) | CAR EDU |
| *Freesia alba* | FRE ALB |  | *Cassine tetragona* (fruit) | CAS TET |
| *Gladiolus cunonius* | GLA CUN |  | *Cynanchum obtusifolium* (fruit) | CYN OBT |
| *Gladiolus exilis* | GLA EXI |  | *Euclea racemosa* (fruit) | EUC RAC |
| *Gladiolus floribundus* | GLA FLO |  | *Muraltia spinosa* (fruit) | MUR SPI |
| *Gladiolus virescens* | GLA VIR |  | *Olea exasperata* (fruit) | OLE EXA |
| *Hesperantha falcata* | HES FAL |  | *Osteospernum moniliferum* (fruit) | OST MON |
| *Ixia micandra* | IXI MIC |  | *Osyris compressa* (fruit, seed) | OSY COM |
| *Moraea fugax* | MOR FUG |  | *Searsia glauca* (fruit) | SEA GLA |
| *Pelargonium dipetalum* | PEL DIP |  | *Searsia lucida* (fruit) | SEA LUC |
| *Pelargonium lobatum* | PEL LOB |  | *Sideroxylon inerme* (fruit) | SID INE |
| *Pelargonium triste* | PEL TRI |  | *Tetragonia decumbens* (veg.) | TET DEC |
| *Rhoicissus digitata* | RHO DIG |  | *Trachyandra ciliata* (veg.) | TRA CIL |
| *Romulea rosea* | ROM ROS |  | *Trachyandra revoluta* (veg.) | TRA REV |
| *Trachyandra ciliata* | TRA CIL |  | *Zygophyllum morgsana* (seed) | ZYG MOR |
| *Trachyandra revoluta* | TRA REV |  |  |  |
| *Tritonia squalida* | TRI SQU |  |  |  |
| *Watsonia fergusoniae* | WAT FER |  |  |  |
| **Renosterveld** |  |  | **Renosterveld** |  |
| *Babiana patulla* | BAB PAT |  | *Carissa bispinosa* (fruit) | CAR BIS |
| *Cyphia digitata* | CYP DIG |  | *Diospyros dichrophylla* (fruit) | DIO DIC |
| *Freesia caryophyllacea* | FRE CAR |  | *Microloma saggitatum* (veg.) | MIC SAG |
| *Freesia leichtlinii* | FRE LEI |  | *Muraltia spinosa* (fruit) | MUR SPI |
| *Pelargonium lobatum* | PEL LOB |  | *Osteospermum moniliferum* (fruit) | OST MON |
| *Pelargonium repaceum* | PEL REP |  | *Osyris compressa* (fruit) | OSY COM |
| *Watsonia alletroides* | WAT ALL |  | *Searsia glauca* (fruit) | SEA GLA |
| *Watsonia meriana* | WAT MER |  | *Sideroxylon inerme* (fruit) | SID INE |
| **Sand Fynbos** |  |  | **Sand Fynbos** |  |
| *Gladiolus guthriei* | GLA GUT |  | *Carpobrotus edulis* (fruit) | CAR EDU |
| *Gladiolus rogersii* | GLA ROG |  | *Diospyros dichrophylla* (fruit) | DIO DIC |
| *Pelargonium triste* | PEL TRI |  | *Osteospernum moniliferum* (fruit) | OST MON |
| *Trachyandra revoluta* | TRA REV |  | *Searsia glauca* (fruit) | SEA GLA |
| *Watsonia fourcadei* | WAT FOU |  | *Trachyandra revoluta* (veg.) | TRA REV |
