# Supplementary figures and images for: Seasonal availability of edible underground and aboveground carbohydrate resources to human foragers on the Cape south coast, South Africa
Source: PeerJ. 2016 Feb 18;4:e1679. doi: 10.7717/peerj.1679 (PMC4768670; doi:10.7717/peerj.1679)

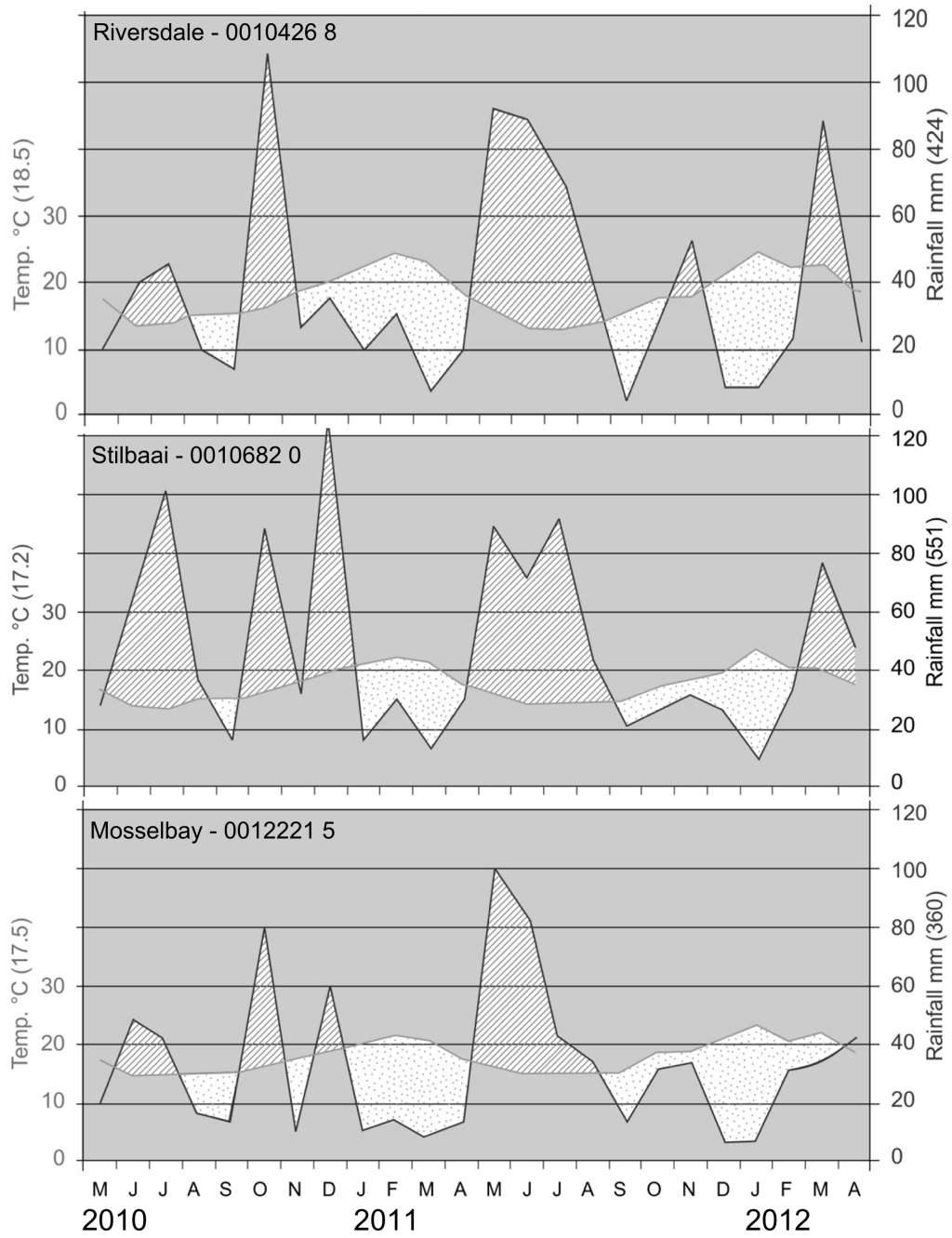

Supplement: Supplemental Information 8 — Climate diagrams showing temperature and rainfall patterns for the study sites during the survey period (May 2010–April 2012). Temperature and rainfall axes and shading follow Walter-Lieth conventions. Mean values of temperature and rainfall for the period are shown in parentheses. The positions of weather stations relative to survey plots are shown in Fig. S2. [file peerj-04-1679-s008.pdf]

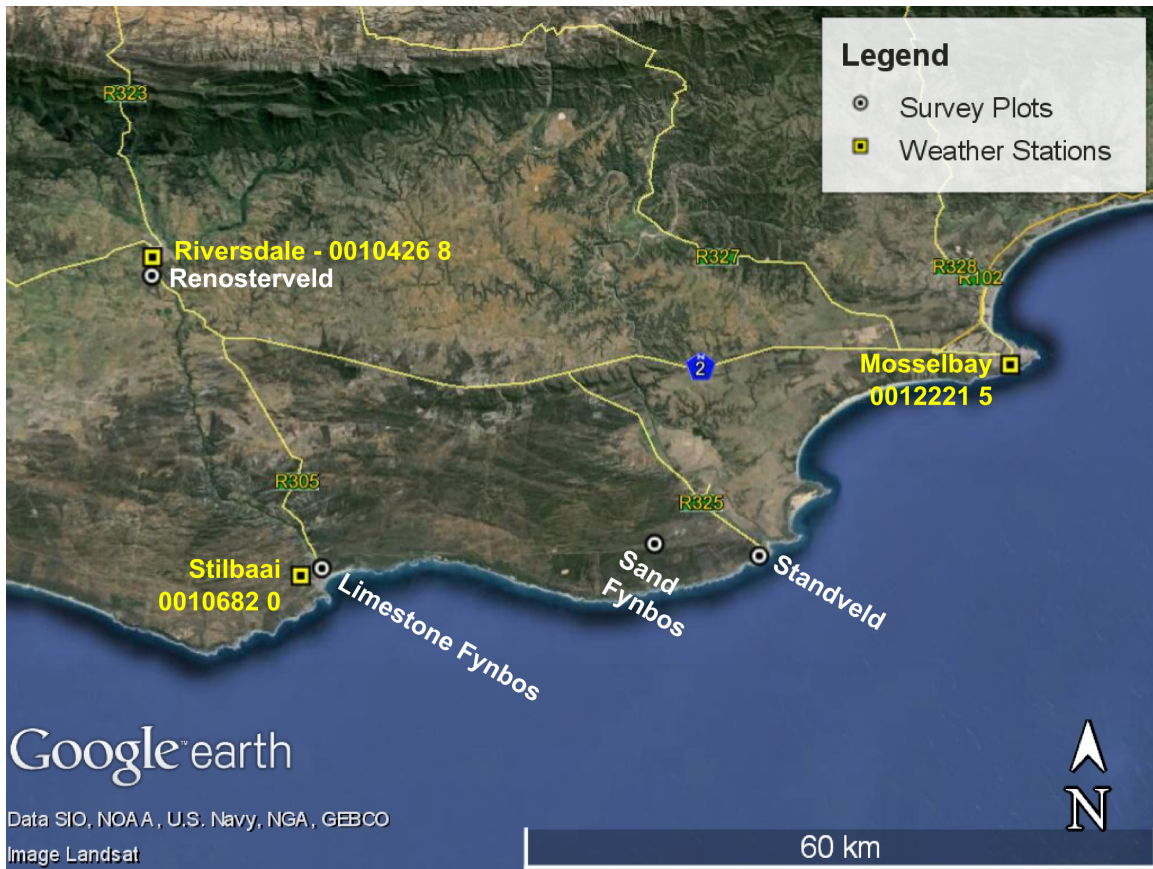

Supplement: Supplemental Information 9 — Locations of the vegetation survey plots and the weather stations (shown in Fig. S1). [file peerj-04-1679-s009.pdf]

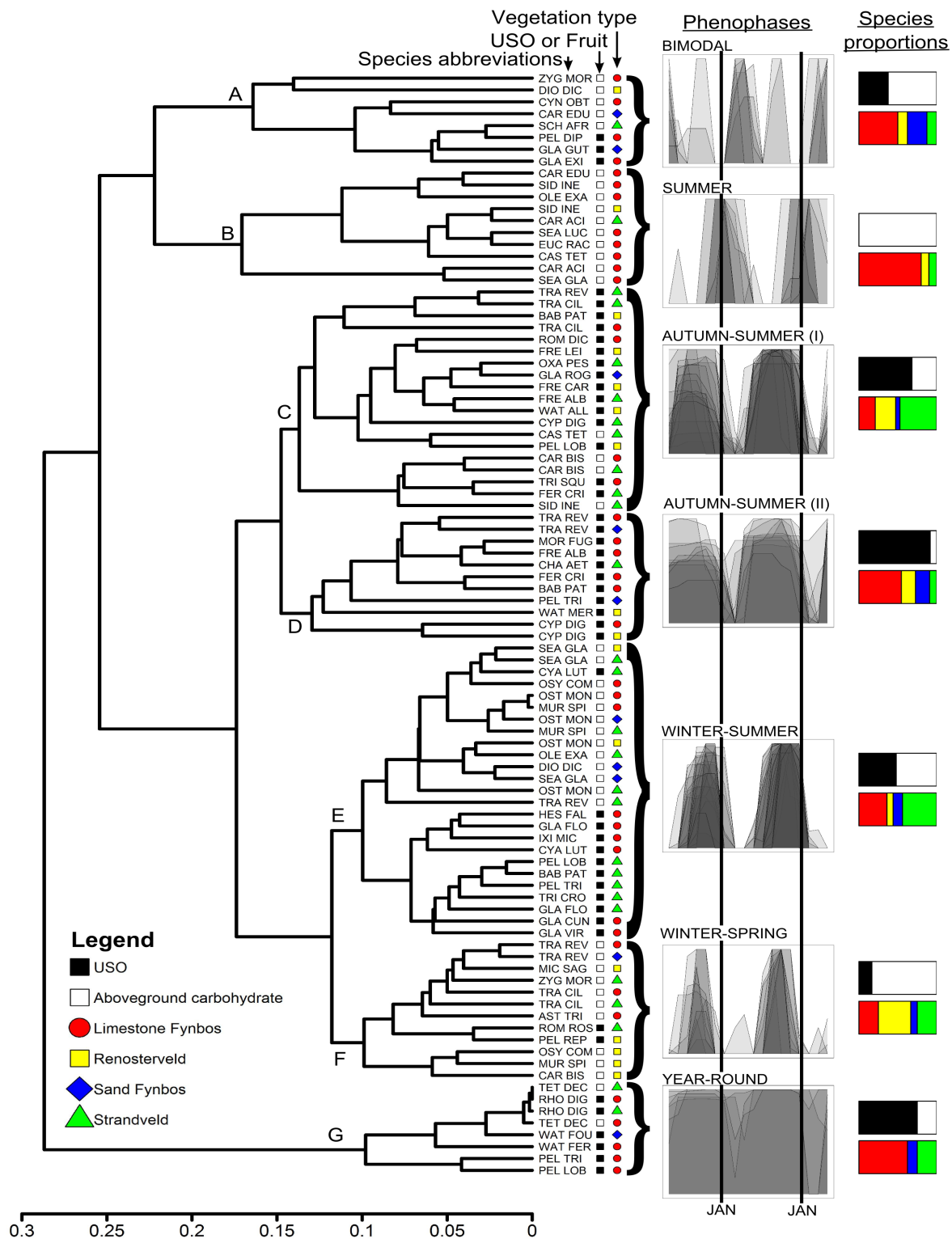

Supplement: Supplemental Information 10 — A hierarchical classification to establish phenological phase synchronicity (specifically availability of edible carbohydrates) among plant species from four different vegetation types along the Cape south coast (South Africa). The plant species, its carbohydrate type (Underground Storage Organ [USO] or aboveground carbohydrate [i.e. fruit, vegetables, seed pods and seeds) and vegetation type are shown. The phenological phase plots show the relative proportion of individuals per species with visible edible carbohydrate through the sampling period within each cluster. The proportion of species within each phenological phase cluster per carbohydrate type (i.e. USO or aboveground) and vegetation type are also shown. Species abbreviations are Tables S3 and S4. See Supplementary Methods for full explanation of the methods used to generate this figure. [file peerj-04-1679-s010.pdf]
